# Kinematic Metrics from a Wireless Stylus Quantify Tremor and Bradykinesia in Parkinson's Disease

**DOI:** 10.1155/2019/6850478

**Published:** 2019-04-02

**Authors:** Andres Maldonado-Naranjo, Mandy Miller Koop, Olivia Hogue, Jay Alberts, Andre Machado

**Affiliations:** ^1^Department of Neurological Surgery, Neurological Institute, Cleveland Clinic Foundation, Cleveland, OH, USA; ^2^Department of Biomedical Engineering, Lerner Research Institute, Cleveland Clinic Foundation, Cleveland, OH, USA; ^3^Department of Quantitative Health Sciences, Lerner Research Institute, Cleveland Clinic Foundation, Cleveland, OH, USA; ^4^Departments of Biomedical Engineering and Center for Neurological Restoration, Neurologic Institute, Cleveland Clinic Foundation, Cleveland, OH, USA; ^5^Departments of Neurological Surgery and Center for Neurological Restoration, Neurologic Institute, Cleveland Clinic Foundation, Cleveland, OH, USA

## Abstract

A fundamental challenge in the clinical care of Parkinson disease (PD) is the current dependence on subjective evaluations of tremor and bradykinesia. New technologies offer the ability to evaluate motor deficits using purely objective measures. The aim of this study was to develop and evaluate the efficacy of a wireless stylus (Cleveland Clinic Stylus) with an embedded motion sensor to quantitatively assess tremor and bradykinesia in patients with PD with subthalamic nucleus (STN) deep brain stimulation (DBS). Twenty-one subjects were tested in various on and off DBS conditions while holding the Cleveland Clinic Stylus while at rest, maintaining a postural hold, and during a movement task. Kinematic metrics were calculated from the motion sensor data, including 3D angular velocity and 3D acceleration data, and were compared between the on and off conditions. Generalized estimating equations (GEEs) were used to determine the relationship between kinematic metrics and MDS-Unified Parkinson's Disease Rating Scale Motor III (UPDRS-III) subscores. Kinematic metrics from the rest and postural tasks were significantly related to the UPDRS-III subscores of tremor (*p* < 0.001 for all metrics), and kinematic metrics from the movement task were significantly related to the UPDRS-III subscore of bradykinesia (*p* < 0.001 for 3/7 metrics). Kinematic metrics (7/9) showed a significant effect of stimulation setting (range: *p* < 0.03– < 0.0001) across the three tasks, indicating significant improvements from DBS in these measures. The Cleveland Clinic Stylus provided quantitative kinematic measures of tremor and bradykinesia severity and detected significant improvements in these measures from medication and DBS therapy. This low-cost, easy-to-use tool can provide objective measures that will improve clinical care of PD patients by providing a more reliable and objective evaluation of movement symptoms, disease progression, and effects of therapy in and outside the clinical setting.

## 1. Introduction

Parkinson's disease (PD) is a movement disorder characterized primarily by tremor, bradykinesia, and rigidity due to dysfunction of the dopaminergic striatal system in the basal ganglia [1, 2]. A fundamental challenge and gap in the clinical care of patients with PD is the current dependence on subjective evaluations of tremor and bradykinesia [3], which in turn is prone to the placebo effect and poor interrater and intrarater reliability for subsections of the MDS-Unified Parkinson's Disease Rating Scale Motor III (UPDRS-III) subscores [4–6]. The exponential increase in computing power, resulting in the development of smaller and more affordable electronics [7], provides the opportunity to utilize technology to objectively evaluate PD.

The integration of inertial measurement units (IMUs) into functional-based instruments provides a mechanism for easy collection of spatial-temporal data that can be used in conjunction with well-established kinematic analyses to objectively quantify specific aspects of motor impairments in PD. In addition to providing an unbiased quantitative assessment of motor performance, these kinematic metrics can be used to identify the impact of physical training, pharmacological, and surgical interventions on daily function [8, 9]. Furthermore, these instruments create the potential to untether movement assessments from the clinical setting and allow for physicians to more precisely track disease progression via self-administered, at-home assessments performed repeatedly throughout the days and weeks between clinical visits.

Our prior work in the use of IMU data from a smart phone to characterize postural stability in healthy young adults [10], older adults [11], and PD patients [12] has demonstrated that smart phone-derived kinematic data provide valid and sensitive measures of gait and postural stability [13, 14]. Leveraging our previous work, we have developed a functional-based motion sensor stylus to quantitatively characterize kinematic changes in PD-related tremor and bradykinesia of upper extremities. The design of the Cleveland Clinic Stylus was developed to create a more ecologically familiar experience for the patient. To achieve the transformative potential of any device that could be used to digitize motor function in PD, the device must be validated in terms of technical capability and its relationship to accepted clinical measures. Therefore, the aim of this project was to determine the relationship between tremor and bradykinesia measures from the Cleveland Clinic Stylus and clinician-rated MDS-UPDRS-III subscores under various deep brain stimulation (DBS) settings that reflect conditions of clinical practice.

## 2. Methods

### 2.1. Subjects

PD subjects with intact cognitive functioning were recruited from the patient population within the Center for Neurological Restoration at the Cleveland Clinic Neurological Institute. All subjects signed an informed consent approved by the Institutional Review Board of the Cleveland Clinic (Cleveland, OH) prior to study participation. All data were collected within the Center for Neurological Restoration. Exclusion criteria were a diagnosis of dementia of any etiology and an inability to understand the study and/or provide informed consent.

All subjects were previously prescribed antiparkinsonian medication and had previously undergone bilateral or unilateral DBS in the subthalamic nucleus for treatment of PD motor symptoms. Medication doses and stimulation parameters were clinically optimized and stable for at least six months prior to study participation. Subjects were tested in the following therapy states: off medication/off DBS (*N*=7), off medication/on DBS (*N*=5), and on medication/on DBS (*N*=16), with some patients tested multiple times in the same conditions and some only once. For assessments including the off medication condition, antiparkinsonian medications were withheld for approximately 12 hours. Assessment including the off-DBS conditions were performed approximately 10–20 minutes after DBS was turned off.

### 2.2. Data Collection

**Figure 1:**
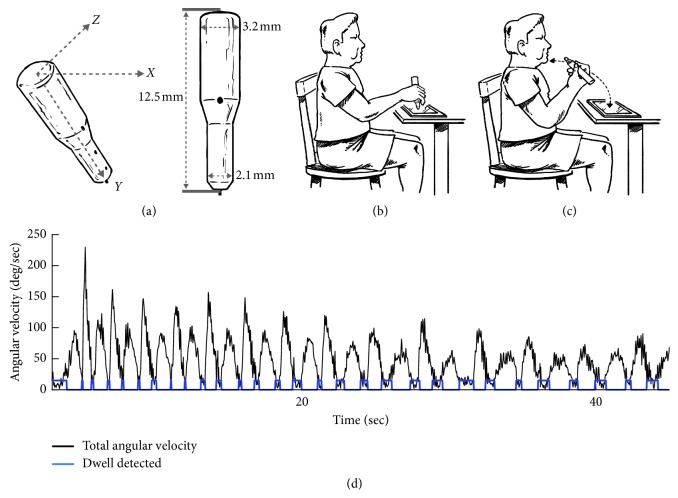
(a–c) Cleveland Clinic Stylus and illustration of test conditions and (d) raw angular velocity (black trace) for a representative subject performing the speed motor test.

Clinical assessment of PD motor symptoms was evaluated using the MDS-UPDRS-III Motor Exam [3], which was administered by the same experienced rater for all subjects. Quantitative assessment of upper extremity tremor and bradykinesia was calculated using an Android tablet, in-house designed software, and instrumented stylus (Figure 1(a)). The instrumented stylus was outfitted with the following components: nine degrees of freedom inertial measurement unit (IMU), Bluetooth modem, and a force-tip displacement. Linear acceleration was measured by a 3-axis linear accelerometer (model ADXL345; ANALOG DEVICE) with a range of ±2 g and a 3-bit resolution at ±16 g. Device rotation rates were measured with a 3-axis gyroscope (ITG-3200 triple-axis MEMS gyroscope) with a range of ±2000 deg/sec and a sensitivity of 14.375 LSBs per/sec. All data were sampled at 50 Hz.

All data from the instrumental stylus were uploaded to the tablet via a customized application that was written to acquire and store data from the stylus and the tablet. After each data collection session, data were downloaded from the tablet device and underwent off-line analysis.

## 3. Experimental Protocol

The three tasks such as (1) *rest—*resting hands on knees, (2) *postural—*holding arms in raised position parallel to floor, and (3) *speed motor task—*repetitive pointing task touching the tip of the stylus to chin and then to an icon on the tablet's screen as fast as possible were performed with the participants seated at a table with their arm in a comfortable writing position (Figures 1(b) and 1(c)). All subjects performed several practice trials. To start each trial, subjects tapped an icon on the screen to initiate a countdown of 5 seconds. After the 5-second interval, an auditory signal was presented that signaled the subject to begin the test. Data were collected for 45 seconds for each of the three tasks per side and were synchronized with the presentation of the auditory signal.

### 3.1. Data Processing and Quantification of PD Motor Symptoms

All off-line analyses were performed using custom MATLAB scripts (MATLAB 9.1.0-R2016b). Angular velocity data from the 3D gyroscope were used to quantify the amount of tremor during the rest and postural tests. Specifically, the raw angular velocity data for each axis of the gyroscope were analyzed separately. For assessment of the tremor, the signals of each axis were filtered with a 4th order high-pass Butterworth filter at 2.5 Hz and a 4th order low-pass Butterworth filter at 12 Hz [15–17]. The amplitude of the tremor in each axis was calculated by taking the root mean square (RMS) value of the filtered angular velocity data [15]. Since the axes of the gyroscope were perpendicular, the total amplitude of the tremor was calculated by first determining the resultant of the three signals [12] and then the RMS of the resultant signal. Prior to finding the resultant signal, the power spectral density (PSD) of each axis was computed using Welch's method, with a 1 sec Hanning sliding window and 50% overlap. The spectral maximum was identified between 0 and –12 Hz, and the frequency of the peak power was determined and used to verify that the primary movement for the tremor analysis was within the tremor-specific frequencies: rest tremor 4–6 Hz and postural tremor 5–9 Hz.

Bradykinesia was assessed using both the angular velocity and linear acceleration data during the speed motor function test. The raw data from each of the three axes were filtered separately. Each signal was filtered with a 4th order high-pass filter at 0.25 Hz and a 4th order low-pass filter at 7 Hz [18]. The total acceleration was then calculated as the resultant of the three components of acceleration, and the total angular velocity was calculated as the resultant of the three components of angular velocity. The RMS of the total angular velocity and total acceleration was used to quantify the average positive amplitude of velocity and acceleration in each trial. The peak value of each signal, peak acceleration, and peak angular velocity were also used to quantify maximal speed and maximal acceleration during the speed motor task.

Deficiencies in movement execution of a continuous, repetitive movement were assessed using the angular velocity data gathered during the speed motor task. Although the task is intended to be a continuous, repetitive movement, previous studies have shown that PD affects the integration of continuous limb movement sequences [19]. Raw angular velocity data from each of the three axes for both sensors were filtered with the same parameters used in the bradykinesia assessment. The total angular velocity was calculated as the resultant of the signals from the three axes. The dwell time (DT) was computed similarly to previously described methods (Figure 1(d)) [19]. In summary, the peak velocity of each movement cycle was found using a peak detection algorithm (MATLAB 9.1.0-R2016b, Chronux Toolbox, and findpeak.m). From the point of each peak velocity, a forward and backward search was performed to find the first point in the angular velocity trace that was 5% of the maximum velocity value for the total trial. The time interval between two peaks of maximum velocity where the value of the angular velocity data was equal to or less than 5% of the maximum value for the entire trial was defined as the dwell time. The DT, coefficient of variation of dwell time (cvDT), and frequency (number of DT/trial) were metrics used to assess the number of completed starting and stopping cycles and the degree to which the stopping and starting motion during the speed motor function test was continuous or discrete in nature.

Clinical measures of upper extremity bradykinesia and tremor were extracted from the MDS-UPDRS-III-motor score. Items 3.4, 3.5, and 3.6 for both the right and left side were calculated for a measure of lateralized bradykinesia and items 3.15 and 3.17 per side for the lateralized tremor.

### 3.2. Variables

The outcome metrics in this study included rest tremor (RMS_rest_ Vel (deg/sec)), postural tremor (RMS_postural_ Vel (deg/sec)), and speed motor task (peak angular velocity (Peak Vel (deg/sec)) and peak linear acceleration (Peak Acc (deg/sec^2^)), RMS angular velocity (RMS_SMT_ Vel (deg/sec)) and linear acceleration (RMS_SMT_ Acc (deg/sec^2^)), Dwell time (DT (sec)), coefficient of variation of DT (cvDT (unitless)), frequency (DT/trial (Hz)), lateralized bradykinesia, and tremor MDS-UPDRS-III subscores).

### 3.3. Statistics

Descriptive statistics were calculated to characterize the sample. A series of generalized estimating equations (GEEs) [20] was used to assess the relationship between MDS-UPDRS tremor and bradykinesia subscores and kinematic measures and to test for significant differences in kinematic measurements between the off and on treatment states. GEEs are a semiparametric regression technique that is appropriate when repeated measurements are taken on each subject, and more common analyses cannot be used because of the violation of the assumption of independence. GEEs allow analyses to address the inherent correlation among multiple responses from the same subjects. GEE adjusts standard regression parameters for response clustering, producing more efficient and unbiased estimates of group-level effects, and can be used with non-Gaussian response variables.

GEE analyses comparing MDS-UPDRS scores and kinematic measures included all observations and regressed each kinematic measure separately on each of the MDS-UPDRS tremor and bradykinesia subscores, while controlling for medication status (on or off) and DBS status (on or off) at the time of assessment. MDS-UPDRS-III tremor and bradykinesia subscores were treated as continuous. Estimates generated provide a measure of the average degree of change in kinematic measure per unit change in MDS-UPDRS measure, while holding medication and stimulation status constant. To address our secondary aim, GEE analysis testing measurements between on and off treatment states included only observations from subjects who were tested in both treatment states and regressed each kinematic measure separately on a binary treatment status variable (on or off medications and DBS). Estimates generated provide a measure of the average difference in kinematic measure between treatment states.

For each GEE, an exchangeable correlation structure was implemented, and empirical standard error estimates are presented. The quasi-likelihood information criterion was used to evaluate model fit. RMS_rest_ Vel, RMS_postural_ Vel, and Peak Acc outcomes were lognormally distributed, and models were built using the log link function. All other outcomes were Gaussian and employed the identity link. Data analyses were conducted using SAS Studio v. 3.5 with an alpha of 0.05.

## 4. Results

Twenty-one subjects (mean age of 65.6 ± 5.0, 14 males) were assessed for a total of 62 observations. Twelve subjects had bilateral implants. Thirty-five (56.45%) observations were conducted in the on medication/on DBS state, 15 (24.19%) in the off medication/off DBS, and 12 (19.35%) in the off medication/on DBS state. Of the 62 observations, the median total MDS-UPDRS-III score was 25 (IQR = 17.5–42, range 8–58) and the median lateralized MDS-UPDRS-III score was 7.5 (IQR = 4.5–12, range 2–21).

### 4.1. MDS-UPDRS III and Kinematic Measures

When adjusting for the nuisance covariance among clustered observations and controlling for medication and DBS state, RMS_rest_ Vel, RMS_postural_ Vel, RMS_SMT_ Acc, RMS_SMT_ Vel, and Peak Acc were significantly associated with the MDS-UPDRS III tremor subscale, while RMS_SMT_ Vel, DT, and frequency were significantly associated with the MDS-UPDRS III bradykinesia subscale. In detail, for every one point increase in the MDS-UPDRS III tremor score, RMS_rest_ Vel increased 88.7% (*p* < 0.0001), RMS_postural_ Vel increased 74.9% (*p* < 0.0001), RMS_SMT_ Acc increased 0.03 m/sec^2^ (*p* < 0.0001), RMS_SMT_ Vel increased 8.26 deg/sec (*p*=0.013), and Peak Acc increased 15.9% (*p*=0.018). For every one point increase in the bradykinesia subscale, RMS_SMT_ Vel decreased 7.3 deg/sec (*p* < 0.0002), dwell increased 0.04 sec (*p* < 0.0009), and frequency decreased 1.86 cycles/sec (*p* < 0.0001). See Table 1 for full details comparing the tremor subscale and all kinematic measures and Table 2 for full details comparing the bradykinesia subscale and all kinematic measures.

### 4.2. Secondary Analysis: Kinematic Measures in Off/On States

From the 21 subjects, 6 subjects (4 males) had both on and off therapy assessments for a total of 26 observations. Five of the subjects had bilateral implants. Median total MDS-UPDRS III score was 10.75 (IQR = 22–47, range 11–58), and median lateralized MDS-UPDRS III score was 10.75 (IQR = 7–12.5, range 3–21).

### 4.3. Group Analysis of Kinematic Measures in Off/On States

When considering the six subjects who completed testing on both off and on medication states and adjusting for the nuisance covariance among clustered observations, all but two kinematic measures displayed significant differences between treatment states. In detail, when on medication/on DBS and comparing to off medication/off DBS, RMS_rest_ Vel scores were 218% lower, RMS_postural_ Vel scores were 175% lower, RMS_SMT_ Acc scores were 0.12 m/sec^2^ faster, RMS_SMT_ Vel scores were 37.65 deg/sec faster, Peak Acc scores were 54% higher, dwell times were 0.12 sec less, and frequency scores increased 11.08 cycles/sec.  Table 3 includes full details comparing the on medication/on DBS state to the off medication/off DBS state.

### 4.4. Example Data from One Subject for Each Quantitative Test

**Figure 2:**
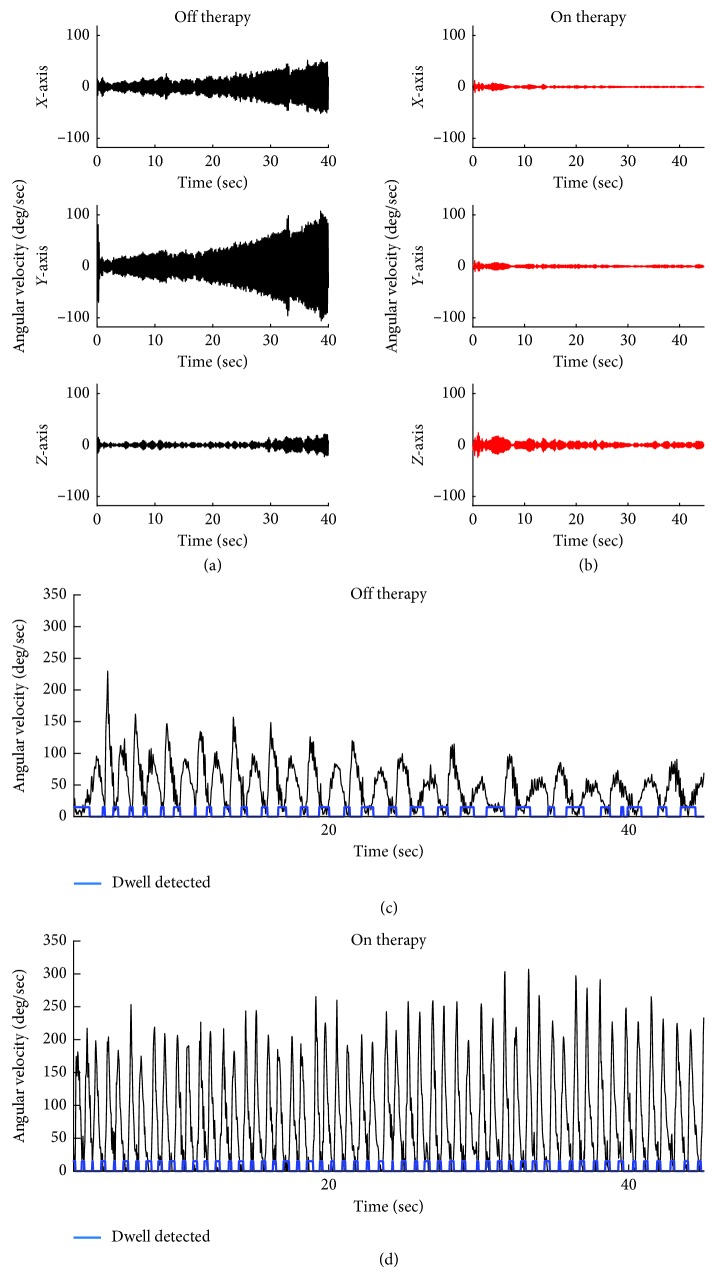
Angular velocity data from each axis of the 3D gyroscope during the rest tremor test off medication/off DBS therapy (a) and on medication/on DBS (therapy) (b) for one representative PD subject. Total angular velocity (black trace) for a representative subject performing the speed motor test off medication/off DBS therapy (c) and on medication/on DBS therapy (d).

Figure 2 (upper panel) shows the 3D angular velocity measurements from a representative subject during the rest tremor protocol during off/off (Figure 2(a)) and on/on (Figure 2(b)) therapy. In off/off condition, rest tremor was present in all three axes, with *X*- (pitch-) axis and *Y*- (roll-) axis showing greater amplitude of tremor than the *Z*- (yaw-) axis. The tremor in all three directions became more severe with time. Total amplitude of the tremor in the off therapy state was 46 deg/sec. In contrast, the amplitude of the rest tremor on therapy was barely visible in the *X*-axis and *Y*-axis, and only a small amount of tremor was seen in the *Z*-axis. Overall, the total amplitude of the tremor for this subject was decreased by 87% in the on/off therapy conditions. These findings were similar for the postural tremor assessments as well.

The lower panel in Figure 2 shows the resultant of the angular velocity data during the speed motor task for one PD subject in the off/off and on/on therapy conditions. The resultant of the angular velocity of the movement is cyclic, with periods of high velocity (peaks) and periods of low or no velocity reflecting times in the trial where the subject was touching a target (chin or tablet). The movement in the off/off therapy condition is overall slower than the on/on conditions and is seen not only in the value of the “peaks” in Figure 2(c) versus Figure 2(d), but is also evident by the number of cycles this subject is able to complete. To perform this repetitive aiming movement well, the subject is required to quickly execute a sequence of starting and stopping limb movements. The dwell time, or time spent on the target, along with the cvDT was used to capture the subject's ability to perform this movement in a continuous fashion versus a sequence of discrete movements. In comparing the on/on versus the off/off conditions, the dwell times were decreased, and were more regular in duration, indicating that the movement was more continuous.

## 5. Discussion

Kinematic outcome metrics from the Cleveland Clinic Stylus were significantly related to clinical measures of tremor and bradykinesia and were able to detect improvements in tremor and upper extremity limb movements associated with medication and DBS therapies in patients with PD. The MDS-UPDRS III is the gold standard for assessing motor function in PD [3]. It might have a role in detecting mild parkinsonian signs per the recent PREDICT-PD study [21]; however, the clinical scale is subjective and may not provide enough resolution to detect subtle, yet important, changes in motor function that may occur when the disease progresses or the treatment is changed. A more precise method of characterizing motor function in response to medication or surgical intervention will enhance tracking disease progression, tuning patient-specific intervention efforts, facilitate patient stratification based on disease status, and can be used in conducting trials for novel therapies. We highlight the latter, as current trials depend on assessments that have limited intra- or interrater reliability and thus add noise to large data sets, increasing the risk for type-II errors. The significant association between RMS_rest_ Vel, RMS_Postural_ Vel, RMS_SMT_ Acc, RMS_SMT_ Vel, and Peak Acc and the MDS-UPDRS III tremor subscale, as well as RMS_SMT_ Vel, dwell time, and frequency with the MDS-UPDRS III bradykinesia subscale in the present study shows that the quantitative kinematic measures of the stylus tool are valid measures of tremor and bradykinesia in PD. These measures are objective and independent of observer bias and provide increased resolution compared to the UPDRS in terms of measurement scale and in terms of isolating the specific aspects of upper extremity movement that is impaired (e.g., dwell periods versus overall limb movement speed).

### 5.1. Kinematics Measures Are Significantly Related to Tremor and Bradykinesia

The present study demonstrates that kinematic measures were significantly associated with UPDRS scores across multiple therapy states. Specifically, RMS_SMT_ Vel measures decreased on average 7.3 deg/sec for every 1 point increase on the bradykinesia subscale (*p*=0.002). This significant association highlights the increased resolution of the kinematic metric to detect changes in the amplitude of PD bradykinesia. For example, although UPDRS scoring would group all possible tremor assessments into 9 bins, a score between 0 and 8, the RMS_SMT_ Vel measure for bradykinesia, ranges between ∼0 and 58 deg/sec for all possible bradykinesia measurements and thus provide a metric with a higher degree of resolution for detecting changes in bradykinesia due to disease progression or changes in therapies. Other kinematic parameters that were significantly related to the clinical examination were RMS_rest_ Vel (<0.0001), RMS_postural_ rest (<0.0001), RMS_SMT_ Acc (<0.0001), RMS_SMT_ Vel (<0.0127), and Peak Acc (0.018) when evaluating tremor and dwell time (0.0009) as well as dwell frequency (<0.0001) when assessing bradykinesia.

Our findings regarding the MDS-UPDRS tremor subscale and RMS_rest_ Vel and RMS_postural_ Vel are in line with previous work by Elble et al. [22], which described a logarithmic relationship between Tremor Rating scale scores and tremor amplitude. Such a relationship, wherein tremor amplitude increases exponentially as a function of the tremor subscale score, supports our assertion that kinematic devices such as our stylus would provide higher resolution when measuring tremor amplitude, particularly among patients who would score in the higher ranges of the MDS-UPDRS tremor subscale.

Though our current study involved a small number of patients, each patient completed the task multiple times. GEEs were used to determine the relationships between the different kinematic parameters and the clinical rating scales by observing all trials performed by all subjects in the different states (on/on, on/off, off/on, and off/off), finding the above-mentioned significant relationships.

The Cleveland Clinic Stylus was able to detect kinematic changes in tremor and bradykinesia as a function of medication status. Furthermore, the device quantifies on a continuous scale, the degree of change of the different parameters studied, allowing for a more detailed evaluation and perhaps the detection of subtle changes that could otherwise be underestimated by gross neurological examination. In this sense, the Cleveland Clinic Stylus provides physiological biomarkers that will allow accurate and objective evaluation of the effects of therapy and will facilitate the interpretation of these effects among the different providers caring for patients with PD.

The present study shows a clear application of a portable and easy-to-use device-software set that allows clinicians to perform kinematics analysis of the motor components of PD in a purely quantitative form. The applications for this device could be extended to the detection of clinical fluctuations of therapy modifications. The stylus could be brought into the operating room where the neurologist could use it to complement their evaluation during intraoperative assessment. Additionally, DBS programming appears to be a useful application of this device, and its validity requires further assessment. Ultimately, the possibility of extending this application to telemedicine and perhaps close-loop systems for remote, or patient-driven programming with real-time feedback, could represent a significant advancement in neuromodulation as well as population-health management.

This study was performed with a relatively small number of PD patients (*N*=21). Further studies incorporating a larger sample size may provide greater insight into other metrics by providing more data and statistical power to detect significant relationships between the MDS-UPDRS-III and measures from the Cleveland Clinic Stylus. In addition, limb kinematics occurs in all axes of space, and one major advantage of this device is the ability to perform measurements along all 3 axes. However, it is not known if other spatial-temporal metrics that have consistently shown significant association with UPDRS such as amplitude of arm movement would be more sensitive in detecting changes in different therapy states. Further analysis of this data set, using more complex algorithms, such as a Kalman filter, would allow for these and other commonly reported spatial-temporal measures of limb movement to be calculated from IMU data. Further studies are necessary to validate this device in the clinical practice.

## 6. Conclusions

Cleveland Clinic Stylus detected changes in tremor and bradykinesia in a purely quantitative manner. The present study shows the utility of a wireless sensor to detect changes in motor function in patients with PD secondary to modification of therapy. We anticipate that this device can be useful in clinical practice and clinical research by adding observer-independent quantitative examination of function to the existing observer-dependent measures. Furthermore, this portable platform can be instrumental to the advancement of distance healthcare in the delivery of movement disorder care.

## Figures and Tables

**Table 1:** GEE estimates and empirical standard errors, regressing kinematic measures on TREMOR subscale, controlling for medication and DBS status.

	Estimate	SE	*Z*	*p*
RMS_rest_ Vel^*∗*^ (%)	0.89	0.14	6.51	<0.0001^*∗∗*^
RMS_postural_ Vel^*∗*^ (%)	0.75	0.06	12.12	<0.0001^*∗∗*^
RMS_SMT_ Acc (m/sec^2^)	0.03	0.01	5.11	<0.0001^*∗∗*^
RMS_SMT_ Vel (m/sec)	8.26	3.31	2.49	0.01^*∗∗*^
Peak Acc^*∗*^ (m/sec^2^)	0.16	0.07	2.37	0.02^*∗∗*^
Peak Vel (m/sec)	28.01	14.91	1.88	0.06
Dwell time (sec)	0.02	0.02	1.18	0.24
Frequency (Hz)	0.28	1.13	0.25	0.80
CV dwell time (%)	0.03	0.04	0.59	0.40

^*∗*^Metric with a logarithmic relationship; ^*∗∗*^significant metric (*p* < 0.05).

**Table 2:** GEE estimates and empirical standard errors, regressing kinematic measures on bradykinesia subscale, controlling for medication and DBS status.

	Estimate	SE	*Z*	*p*
RMS_rest_ Vel^*∗*^ (%)	6.94	14.01	0.50	0.62
RMS_postural_ Vel^*∗*^ (%)	3.43	70.83	0.30	0.76
RMS_SMT_ Acc (m/sec^2^)	−0.135	0.01	−1.63	0.10
RMS_SMT_ Vel (m/sec)	−7.30	1.98	−3.68	0.0002^*∗∗*^
Peak Acc^*∗*^ (m/sec^2^)	−10.75	6.18	−1.74	0.08
Peak Vel (m/sec)	−13.60	9.35	−1.45	0.15
Dwell time (sec)	0.04	0.01	3.33	0.0009^*∗∗*^
Frequency (Hz)	−1.86	0.48	−3.89	0.0001^*∗∗*^
CV dwell time (%)	−0.04	0.03	−1.53	0.013

^*∗*^Metric with a logarithmic relationship; ^*∗∗*^significant metric (*p* < 0.05).

**Table 3:** GEE estimates, mean values, and empirical standard errors, comparing on/on kinematic measures to off/off kinematic measures.

	DBS off LS mean (SE)	DBS on LS mean (SE)	Difference in estimate (SE)	*Z*	*p*
RMS_rest_ Vel^*∗*^ (%)	320.49 (0.62)	102.65 (0.26)	−217.83 (56.99)	−3.82	0.0001^*∗∗*^
RMS_postural_ Vel^*∗*^ (%)	296.21 (0.52)	1.21 (0.32)	−174.79 (25.02)	−6.94	<0.0001^*∗∗*^
RMS_SMT_ Acc (m/sec^2^)	0.3875 (0.03)	0.5029 (0.05)	0.12 (0.04)	2.74	0.006^*∗∗*^
RMS_SMT_ Vel (m/sec)	105.62 (13.2)	143.28 (14.52)	37.65 (12.71)	2.96	0.003^*∗∗*^
Peak Acc^*∗*^ (m/sec^2^)	0.2147 (0.11)	0.75 (0.23)	0.54 (0.24)	2.24	0.025^*∗∗*^
Peak Vel (m/sec)	346.66 (50.59)	433.73 (50.95)	87.07 (53.12)	1.64	0.101
Dwell time (sec)	0.45 (0.06)	0.33 (0.05)	−0.1231 (0.05)	−2.36	0.018^*∗∗*^
Frequency (Hz)	36.95 (2.46)	48.02 (3.92)	11.08 (3.70)	3.00	0.003^*∗∗*^
CV dwell time	0.98 (0.12)	0.84 (0.09)	−0.13 (0.089)	−1.45	0.146

^*∗*^Metric with a logarithmic relationship; ^*∗∗*^significant metric (*p* < 0.05).

## Data Availability

The clinical and kinematic data used to support the findings of this study may be released upon application to the Cleveland Clinic Institutional Review Board, by contacting Ruth Fritskey at 216-444-2924, IRB@ccf.org.
